# OR2AT4 and OR1A2 counterregulate molecular pathophysiological processes of steroid-resistant inflammatory lung diseases in human alveolar macrophages

**DOI:** 10.1186/s10020-022-00572-8

**Published:** 2022-12-12

**Authors:** Daniel Weidinger, Kaschin Jamal Jameel, Desiree Alisch, Julian Jacobsen, Paul Bürger, Matthias Ruhe, Faisal Yusuf, Simon Rohde, Klemens Störtkuhl, Peter Kaufmann, Juliane Kronsbein, Marcus Peters, Hanns Hatt, Nikolaos Giannakis, Jürgen Knobloch

**Affiliations:** 1 Medical Clinic III for Pneumology, Allergology and Sleep Medicine, Bergmannsheil University Hospital, Ruhr-University Bochum, Bürkle-de-la-Camp-Platz 1, 44789 Bochum, Germany; 2 AG Physiology of Senses, Ruhr-University Bochum, Universitätsstraße 150, 44801 Bochum, Germany; 3 Department of Molecular Immunology, Ruhr-University Bochum, Universitätsstraße 150, 44801 Bochum, Germany; 4 Department of Cell Physiology, Ruhr-University Bochum, Universitätsstraße 150, 44801 Bochum, Germany

**Keywords:** Alveolar macrophages, Olfactory receptor, Therapeutic target, Obstructive lung diseases

## Abstract

**Background:**

Therapeutic options for steroid-resistant non-type 2 inflammation in obstructive lung diseases are lacking. Alveolar macrophages are central in the progression of these diseases by releasing proinflammatory cytokines, making them promising targets for new therapeutic approaches. Extra nasal expressed olfactory receptors (ORs) mediate various cellular processes, but clinical data are lacking. This work investigates whether ORs in human primary alveolar macrophages could impact pathophysiological processes and could be considered as therapeutic targets.

**Methods:**

Human primary alveolar macrophages were isolated from bronchoalveolar lavages of 50 patients with pulmonary diseases. The expression of ORs was validated using RT-PCR, immunocytochemical staining, and Western blot. Changes in intracellular calcium levels were analyzed in real-time by calcium imaging. A luminescent assay was used to measure the cAMP concentration after OR stimulation. Cytokine secretion was measured in cell supernatants 24 h after stimulation by ELISA. Phagocytic ability was measured by the uptake of fluorescent-labeled beads by flow cytometry.

**Results:**

We demonstrated the expression of functional OR2AT4 and OR1A2 on mRNA and protein levels. Both ORs were primarily located in the plasma membrane. Stimulation with Sandalore, the ligand of OR2AT4, and Citronellal, the ligand of OR1A2, triggered a transient increase of intracellular calcium and cAMP. In the case of Sandalore, this calcium increase was based on a cAMP-dependent signaling pathway. Stimulation of alveolar macrophages with Sandalore and Citronellal reduced phagocytic capacity and release of proinflammatory cytokines.

**Conclusion:**

These are the first indications for utilizing olfactory receptors as therapeutic target molecules in treating steroid-resistant lung diseases with non-type 2 inflammation.

**Supplementary Information:**

The online version contains supplementary material available at 10.1186/s10020-022-00572-8.

## Background

Chronic lung diseases affect over 10% of the worldwide population (Enilari and Sinha 2019; Soriano et al. 2020). They include asthma, chronic obstructive pulmonary disease (COPD), cystic fibrosis, and others (Boucher 2019; Osadnik and Singh 2019). The underlying causes vary between diseases and phenotypes and have genetic components and environmental triggers such as direct exposure to pollutants (Beasley et al. 2015; Cutting 2015; Quaderi and Hurst 2018). Obstructive lung diseases share symptoms, such as increased mucus production, airway obstruction due to tissue alterations, and a proinflammatory environment induced by increased cytokine production in the affected lung areas (Wang et al. 2020; Chen et al. 2021; Lara-Reyna et al. 2020). If the local inflammation becomes persistent, it might cause lung tissue remodeling processes, resulting in irreversible damage and loss of lung function (Wang et al. 2018; Hough et al. 2020; Castellani et al. 2018).

The majority of inflammations in phenotypes of obstructive lung diseases are classified as non-type 2 or type 2, depending on the contribution of different immune cell types and cytokines. Type 2 inflammation is key in several asthma phenotypes (Fahy 2015). Type 2 inflammation is mainly sensitive to inhaled steroids and/or can be reduced with biologics, targeting the key cytokines or cytokine receptors, e.g., in severe asthma (Licari et al. 2020; Matucci et al. 2021). In contrast, anti-inflammatory treatment of diseases with non-type 2 inflammation, like COPD, phenotypes of non-type 2 asthma (Duvall et al. 2019), or cystic fibrosis is limited because of the partial or complete resistance to inhaled corticosteroids and the lack of appropriate biologics (Barnes 2009; Mei et al. 2019; Adcock et al. 2008). Therefore, the current treatment of such disease phenotypes is primarily symptomatic but not causal and can not stop or substantially reduce disease progression (Garth et al. 2018).

Alveolar macrophages (AM) are central in non-type 2 inflammation. In AM, hyperactivation of transcription factors such as NF-κB or AP-1 results in the secretion of various proinflammatory cytokines such as interleukin-6 (IL-6), chemokine (CXC motif) ligand 8 (CXCL-8, IL-8), chemokine (CC motif) ligand 2 (CCL-2), or matrix metallopeptidase 9 (MMP-9) (Hikichi et al. 2019; Khanjani et al. 2012; Liu et al. 2018; Schwartz et al. 1996), all of which promote local non-type 2 inflammation and, therefore, disease progression.

Inflammation and disease progression of non-type 2 respiratory diseases might be triggered not only by defects in cytokine secretion (Kapellos et al. 2018; Lambrecht and Hammad 2013; Stecenko et al. 2001), but also by defects in the intracellular calcium balance (Rimessi et al. 2021; Petit et al. 2019) and by phagocytosis (Belchamber and Donnelly 2017). Patients with non-type 2 inflammation are more susceptible to respiratory infections (Knobloch et al. 2011a, 2019; Jartti et al. 2020; Kiedrowski and Bomberger 2018), leading to a further increase in inflammation and finally to exacerbations (Stolz et al. 2019). There is an indication at least for COPD that the increased susceptibility might be caused by a reduced production of cytokines required for infection defense by systemic immune cells during their recruitment and activation processes (Knobloch et al. 2011a, 2019). However, the CXCL-8 and MMP-9 hyperproduction of local and resident AM in response to infectious trigger might contribute to the deleterious increase in inflammation which causes exacerbations (Knobloch et al. 2011b; Culpitt et al. 2003). These molecular pathologies might be considered by designing the urgently required new therapeutic approaches for lung diseases with non-type 2 inflammation.

We hypothesized that ectopic olfactory receptors (ORs) in AM might have potential as drug targets for lung diseases with non-type 2 inflammation. They represent the largest subgroup of G protein-coupled receptors (GPCRs). GPCRs are targets of about one-third of all registered drugs, but the OR subgroup does not yet play a role in this context (Sriram and Insel 2018; Hauser et al. 2017). The latter is surprising because ORs are not only expressed in the nasal epithelium but occur almost ubiquitously (Maßberg and Hatt 2018; Flegel et al. 2013). ORs mediate a wide range of cellular processes that are key for pathophysiologies of chronic diseases like proliferation (Weidinger et al. 2021; Chéret et al. 2018), apoptosis (Weber et al. 2017), migration (Jovancevic et al. 2017; Weber et al. 2018), and hormone regulation (Braun et al. 2007). ORs also influence the calcium balance of certain cell types (Kalbe et al. 2017), which is misregulated in various diseases and might be considered a therapeutic target (Rimessi et al. 2020; Chiu et al. 2017). The responses to the stimulation of a specific OR can differ across multiple cell types. Stimulation of OR2AT4 with its ligand Sandalore increased proliferation in keratinocytes but also reduced proliferation and induced apoptosis in myelogenous leukemia cells (Busse et al. 2014; Manteniotis et al. 2016a). Because of the large number of different ORs and characterized agonists and antagonists there might be many drug target candidates. The cellular processes that are influenced by different ORs can differ in the same cell type. For example, Kalbe et al. showed that activation of OR1D2 led to an enhanced contraction of airway smooth muscle cells, which was not observed upon stimulation of OR2AG1 (Kalbe et al. 2016). Therefore, it is necessary to characterize the effects of individual ORs in the target cell type as a prerequisite for developing drug target strategies.

This study aimed to investigate whether ectopically expressed ORs impact pathophysiological processes in alveolar macrophages, a cell type with key functions in obstructive lung diseases with steroid-resistant non-type 2 inflammation. With this, we aimed to investigate our hypothesis that ORs are drug target candidates for causal therapies in such phenotypes of obstructive lung diseases.

## Methods

### Fiberoptic bronchoscopy

The fiberoptic bronchoscopy and the isolation of the bronchoalveolar lavage (BAL) were performed as described before (Knobloch et al. 2011b; Koch et al. 2004). Briefly, 60 ml pre-warmed 0.9% NaCl solution was applied four times to the middle lobe and then aspirated. From this, 100–150 ml bronchoalveolar lavage fluid was obtained.

### Isolation of alveolar macrophages from the bronchoalveolar lavage fluid

Alveolar macrophages (AM) were isolated from the BAL as previously described (Knobloch et al. 2011b; Koch et al. 2004), with minor modifications. To remove mucus plugs, the BAL was filtered through a 70 µM nylon strainer (Thermo Fisher Scientific, Waltham, USA, cat: 352350). The fluid was filled up to a total volume of 50 ml using Ca^2+^/Mg^2+^-free phosphate-buffered saline (PBS; Thermo Fisher Scientific, cat: 14200083) and was centrifuged for 10 min at 450 xg at 4 °C. The supernatant was removed, and the pellet was washed with 50 ml PBS. After a further centrifugation step, the pellet was resuspended in culture medium, Roswell Park Memorial Institute 1640 (RPMI; Sigma-Aldrich, St. Louis, USA, cat: R7638) containing: 10% (w/v) fetal bovine serum (Pan Biotech, Aidenbach, Germany, cat: P30-1506), 100 U/ml Penicillin, 100 µg/ml Streptomycin (Sigma-Aldrich, cat: P4333), 2 mM l-glutamine (Sigma-Aldrich, cat: G7513) and 0.25 µg/ml Amphotericin B (Sigma-Aldrich, cat: A2942). Cell numbers were determined using a Neubauer hemocytometer. The vitality was analyzed via trypan blue (Sigma-Aldrich, cat: T8154) staining. The sample was discarded if the cell death quote was ≥ 10%. For all experiments, AM were seeded in culture vessels and maintained at 37 °C and 5% carbon dioxide (v/v) in a humidified atmosphere. Non-adhered cells were removed by replacing the culture medium before the experiments. According to previous studies (Knobloch et al. 2011b; Gerlach et al. 2015), the purity of the isolated AM was evaluated by morphological analysis and was > 99% in the cultures.

### Patient metrics

In this proof of principle study, alveolar macrophages were isolated from the BAL of 49 patients with lung diseases and/or smoking status that indicate for partial or exclusive non-type 2 inflammation (13 female, 37 male, age: 65.6 ± 1.77 years; Table 1). Due to the lack of donors, one additional subject without indication for any kind of inflammation was included exclusively in the experiment shown in Additional file 1: Fig. S1. The study was carried out according to the Code of Ethics of the World Medical Association. All patients signed their informed consent for the procedure and the use of their samples. The study was approved by the Ethics Committee of the Ruhr-University Bochum (4257-12). Exclusion criteria were an age of < 18 and documented active or preexisting alcohol abuse. Donors were randomly selected for the experiments. Detailed information on the patients used for each experiment can be found in the supplement (Additional file 1: Table S1).

**Table 1:** Patient metrics

Disease	Number of patients (multiple diseases possible)
COPD (Gold I–IV)	17
Other interstitial lung diseases	10
Asthma (mild-severe, smokers)	4
Pneumonia	6
Lung cancer (NSCLC, SCLC)	6
Space-occupying lesions	6
Pulmonary nodules	2
Alveolitis	2
Chronic cough	1
Asthma-COPD overlap syndrome	1
Chronic bronchitis	1
Dyspnea	1
Pulmonary hypertension	1

### RNA isolation and reverse transcriptase PCR

For RNA isolation, 1 × 10^6^ AM were seeded in a 35 mm dish as described above. After 2 h of incubation, the medium was removed, and the cells were rinsed with 1 ml PBS. AM were harvested with a cell scraper in 1 ml fresh PBS, pelleted by centrifugation at 800×*g* for 3 min, and frozen in liquid nitrogen and stored at − 80 °C. RNA extraction was performed using the RNeasy Plus Mini Kit (Qiagen, Hilden, Germany cat: 74134) according to the manufacturer’s protocol with the gDNA Eliminator spin columns and optional centrifugation for 1 min at 18,000×*g*. Complementary DNA (cDNA) was synthesized using the iScript™ cDNA Synthesis Kit (Bio-Rad, Hercules, USA cat: 1708890) according to the manufacturer’s manual. 100 ng RNA was used for synthesis. Genomic DNA contamination was excluded by performing a control approach without adding reverse transcriptase (-RT). The HotStarTaq DNA Polymerase Kit (Qiagen, cat: 203203) was used to perform the reverse transcriptase PCR (RT-PCR). The specific primers used and listed below were designed using the software Primer 3 (Kõressaar et al. 2018; Untergasser et al. 2012) (Version 4.1) and were synthesized by Thermo Fisher Scientific: OR2AT4, forward 5′ GCCCATCCCAGCAGTAGTAAG-3′, reverse 5′ GAGGGGGTTGAGAATTGGTGT-3′; OR1A1: forward 5′ GGAAAATAACCAGTCCTCTACAC-3′, reverse 5′ GAAGACTGTGGAGAAGACTCG-3′; OR1A2: forward 5′ GGCAACCAGGAAGTAGCCAA-3′, reverse 5′ CAGGTGCAGAAGGCTTTGAAT-3′; TATA-Box Binding Protein (TBP, housekeeping gene used for reference): forward 5′ GGGCACCACTCCACTGTATC-3′; reverse 5′ CGTGGTTCGTGGCTCTCTTA-3′. The following temperature cycle profile was used: 15 min at 95 °C, followed by 40 cycles of 30 s at 95 °C, 30 s at 60 °C, 60 s at 72 °C, and a final elongation of 10 min at 72 °C. The PCR products were analyzed using a 1.5% agarose gel containing 1× ROTI GelStain Red (Carl Roth, Karlsruhe, Germany, cat: 0984.1). The size of the synthesized DNA fragments was determined using the GenRuler 100 bp DNA Ladder (Thermo Fisher Scientific, cat: SM0241).

### Western blot

AM were prepared as described above. The cell pellet was dissolved in RIPA buffer (150 mM NaCl, 50 mM Tris, 1% Nonidet, 0.1% SDS, 0.5% Natrium Deoxycholat) with an protease inhibitor added according to the instructions of the manufacturer (Merck, Darmstadt, Germany, cat: 11836153001), fourfold concentrated laemmli (40% Glycerol, 240 mM Tris–Cl, 8% SDS and 0.1% Bromphenolblau) and DTT (5 mM) at a concentration of 30 µl/0.5 × 10^6^ cells. SDS-PAGE and Western blot experiments were performed as previously described (Gelis et al. 2016). The following primary antibodies were used: custom-made affinity purified rabbit IgG polyclonal antibodies against OR1A2 or OR2AT4 (Eurogentec, Seraing, Belgium). The specificity of both antibodies has been shown before by detecting heterologous expressed rho-tagged ORs in Hanna3A cells (Busse et al. 2014; Maßberg et al. 2015). For validation of our results we used a commercially available anti-human-OR2AT4 polyclonal antibody (Thermo Fisher Scientific cat: PA5-71599) and a comercially available anti-human-OR1A2 polyclonal antibody (Thermo Fisher Scientific cat: PA5-99907).

### Immunocytochemical staining

2 × 10^5^ AM were seeded onto a 12 mm diameter cover glass in a 24 well plate containing 500 µl RMPI 1640 with all supplements as described above. After adherence of the AM, the media was removed, and the cells were washed once with PBS. Fixation was done by a 20 min incubation in 4% paraformaldehyde (PFA) at 4 °C. Afterwards, the PFA was removed and the AM were permeabilized by washing three times with PBS + 0.05% Triton X-100 (PBST) for 10 min at room temperature. Unspecific epitopes were blocked by incubation with 10% goat serum diluted in PBST for 1 h. The custom-made primary antibodies against OR2AT4 and OR1A2 were diluted at 1:100 in PBST containing 5% goat serum, whereas the antibody against E-cadherin (Thermo Fisher Scientific cat: 13-1700) was diluted 1:2000. Incubation was done overnight at 4 °C. Cells were washed three times and were then incubated with goat anti-rabbit antibodies Alexa Fluor™ 546 (1:1000, Thermo Fisher Scientific, cat: A-11010), goat anti-mouse Alexa Fluor™ 488 (1:1000, Thermo Fisher Scientific, cat: A-11029), and Hoechst 33342 (1:1000, Thermo Fisher Scientific, cat: 62249), for 45 min in PBST + 5% goat serum at room temperature. The cells were washed with PBST and mounted on a slide with ProLong™ Gold Antifade Mountant (Thermo Fisher Scientific, cat: P36934).

### Odorants

The odorants Sandalore (Symrise, Holzminden, Germany), Brahmanol (Symrise), Oxyphenylon (Henkel, Düsseldorf, Germany), and (±) Citronellal (Sigma-Aldrich, St. Louis, Cat: 373753) were diluted 1:10 in DMSO. A maximal concentration of ≤ 0.2% DMSO was maintained in all experiments.

### Single-cell calcium imaging

A minimum of 1 × 10^5^ AM were plated in a 35 mm dish and incubated for 2 h. The cells were prepared by incubation for 30 min in 1 ml loading buffer containing Ringer solution (140 mM NaCl, 5 mM KCl, 5 mM CaCl_2_, 2.5 mM MgCl_2_, 1 mM HEPES) and 7.5 μM Fura-2 AM (Enzo, Farmingdale, USA, cat. ENZ-52006) like described before (Gelis et al. 2016). After removal of the extracellular Fura-2 AM by washing with Ringer solution, Ca^2+^-ratiometric imaging was performed using a light source (EL6000, Leica), a Leica inverted confocal microscope (DMI6000 CS, Leica) with a 20× objective (UPLSAPO, Olympus, Tokyo, Japan). The images were recorded at 1 Hz by the DFC360 FX (Leica, Wetzlar, Germany), and the integrated fluorescence (***f*** 340 nm/***f*** 380 nm) of each cell was measured using the Leica Application Suite Advanced Fluorescence (LAS AF). The odorants were pre-diluted in DMSO and then adjusted to the final concentration in Ringer solution (140 mM NaCl, 5 mM KCl, 2 mM CaCl_2_, 1 mM MgCL_2_, and 10 mM Hepes). The inhibitors MDL-12,330A (10 µM; Sigma-Aldrich, St. Louis, USA, cat: M182) and Oxyphenylon (300 µM; Henkel) were also pre-diluted in DMSO. EGTA (10 mM; Sigma-Aldrich, St. Louis, USA, cat: 03777) was prepared in water.

### cAMP assay

To analyze the intracellular cAMP concentration, 1 × 10^5^ cells were cultured overnight. Then they were stimulated for 20 min with Sandalore or Citronellal in different concentrations. The cAMP concentration was determined by using cAMP-Glo™ Assay (Promega, Wisconsin, USA, cat: V1501) according to the manufacturer’s protocol.

### AM stimulation

1 × 10^5^ cells were cultured in 100 µl media supplemented as described above for 3 h before serum-withdrawal overnight in medium with 2% FBS. After incubation, 1 × 10^5^ cells were stimulated with Sandalore, Citronellal (each at 10–500 µM), Lipopolysaccharide (LPS, 1 µg/ml, from *Salmonella enteritidis*, Sigma Aldrich, cat: L7770), Lipoteichoic acid (LTA, 10 µg/ml, from *Staphylococcus aureus*, Invivo Gen, San Diego, USA cat:tlrl-slta), and/or peptidoglycan (PGN, 10 µg/ml, from *Staphylococcus aureus*, Invivo Gen cat: tlrl-pgns2) in serum-deprived media. The solvent DMSO (0.1% v/v) served as the control. After 24 h, the supernatants were collected and stored at − 20 °C.

### ELISA

The concentrations of IL-6, CCL-2, CXCL-8, and MMP-9 were measured in supernatants via ELISA (Thermo Fisher Scientific, cat: 88-7066-88, 88-8086-88, 88-7399-88, R&D systems, Minneapolis, USA, cat: DY217B, DY911) according to the manufacturer’s protocols.

### Latex beads phagocytosis assay

Phagocytic activity was analyzed by seeding 1.5 × 10^6^ cells in a Petri dish. After overnight incubation at 37 °C in the incubator, the cells were rinsed twice using PBS. Afterward, AM were detached with trypsin. The reaction was stopped by adding 3 ml of culture medium. The cells were centrifuged at 450×*g* for 8 min. The supernatant was removed and the cells were resuspended in 1 ml of culture medium. The suspension was adjusted to a concentration of 1 × 10^5^ cells per milliliter culture medium. 20 µl Fluoresbrite® YG Microspheres (0.5 µM; Polysciences Inc, Warrington, USA, cat: 17152-10), Sandalore, Citronellal (10–500 µM), or 0.1% DMSO was added to 1 × 10^5^ cells per milliliter. After 2 h of incubation, the reaction was stopped by adding 2 ml PBS. The AM were centrifuged, the supernatants were discarded, and the pellets were fixed in 1.5% PFA. The mean fluorescence intensity of the cells was measured using the Cyflow SL (Partec, Münster, Germany).

### Statistical analyses

Statistical analyses were performed to examine the effects of inhibitors, stimulants, and OR stimulations on calcium balance, phagocytic activity, and secretion of cytokines in AM. The results were normalized to the corresponding controls and are shown as mean ± SEM. The Friedman test with post hoc “Two-stage-up method of Benjamini, Krieger, and Yekutieli” test was used in concentration–response experiments. Cytokine production in response to PAMPs were analyzed with one-sample t-tests. For the analysis of inhibitor effects, the paired t-test was used. GraphPad Prism 9 was used for statistical analysis.

## Results

### Sandalore, Citronellal, and Brahmanol all increased the intracellular calcium levels of AM

**Fig. 1:**
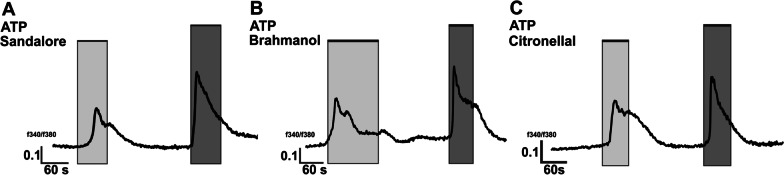
Stimulation of AM with Sandalore (**A**), Brahmanol (**B**), or Citronellal (**C**) increases intracellular calcium. The application period of the odorant (300 µM) is marked in light grey ATP (100 µM) was applied as a vitality control (dark grey)

To identify functionally expressed ORs in primary human AM, Ca^2+^ imaging was used because previous studies showed changes in intracellular calcium concentrations after stimulation of ectopically expressed ORs (Jovancevic et al. 2017; Maßberg et al. 2015; Manteniotis et al. 2016b). We tested five known agonists for commonly expressed ORs (Helional, Sandalore, Brahmanol, Citronellal, and Citronellol) as well as two OR agonists secreted by respiratory bacteria (Methylcyclohexane and Heptane) (Abd El Qader et al. 2015). AM responded to three of them: Sandalore, Brahmanol, and Citronellal. All increased intracellular calcium concentrations (Fig. 1). Sandalore and Brahmanol are ligands of OR2AT4, while Citronellal is a ligand of both OR1A1 and OR1A2 (Busse et al. 2014; Maßberg et al. 2015; Schmiedeberg et al. 2007).

### Proteins of OR2AT4 and OR1A2 are functionally expressed in AM

**Fig. 2:**
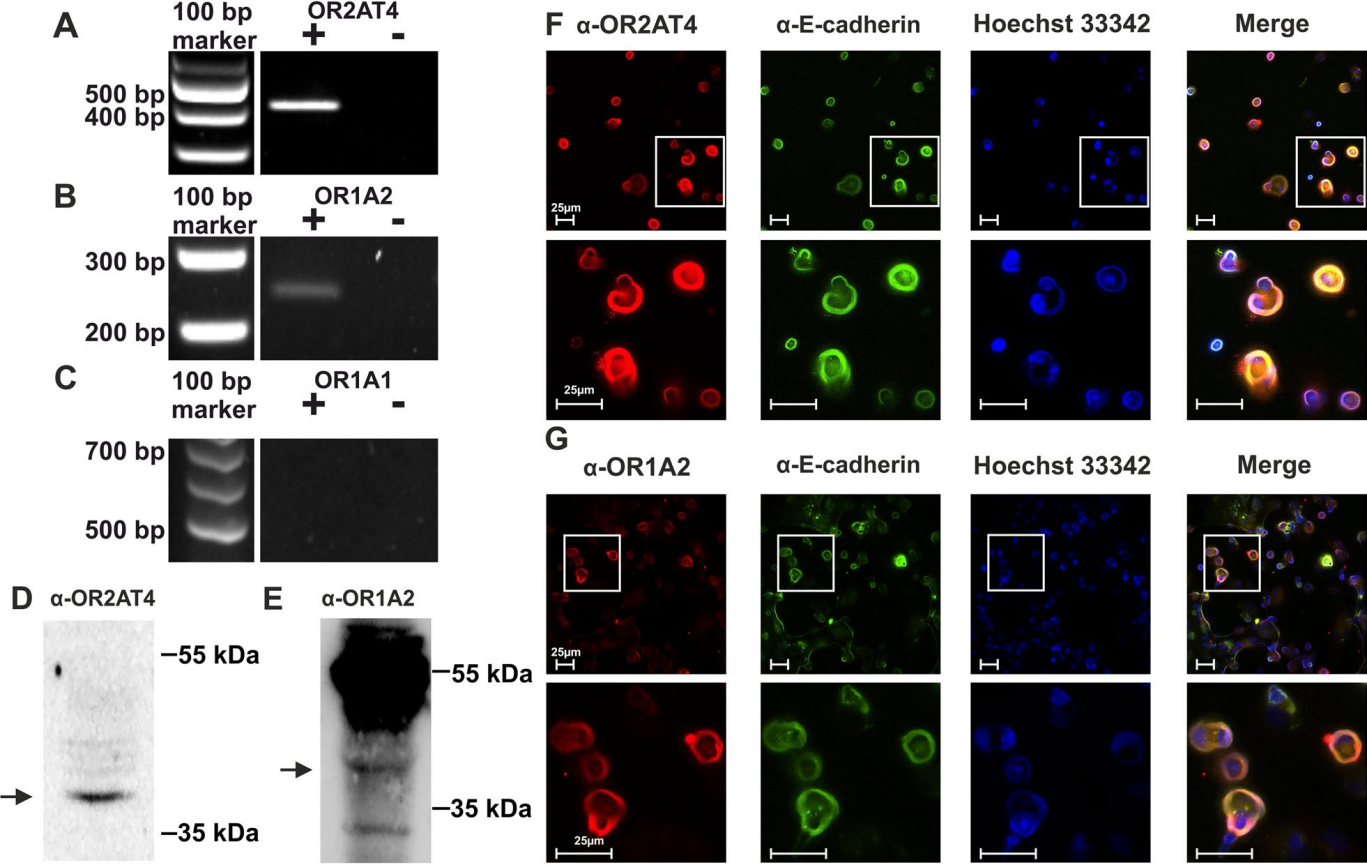
OR2AT4 and OR1A2 are expressed in alveolar macrophages. Transcripts of OR2AT4 (**A**), OR1A2 (**B**), and OR1A1 (**C**) were analyzed by RT-PCR. “+” or “−“ indicate the presence and absence (negative control) of the reverse transcriptase in the enzyme mix. The estimated sizes of OR2AT4, OR1A2, or OR1A1 were 400, 212, or 670 bp (n = 3). OR2AT4 (**D**) and OR1A2 (**E**) proteins were detected by Western blot (n = 2–3). The estimated size of both OR2AT4 and OR1A2 was 34 kDa (arrows). **F**, **G** Intracellular localization of OR2AT4 and OR1A2 (each in red) by immunocytochemistry (n = 3). The plasma membrane was stained by an antibody against E-cadherin (green). Hoechst 33,342 marked the nucleus (blue). The merge shows the overlay of all markers. Bars indicate a range of 25 µm and squares mark the zoomed area. (n = 3)

The expression of the associated receptors OR2AT4, OR1A1, and OR1A2 was investigated. The mRNAs of OR2AT4 and OR1A2 were detected by RT-PCR (Fig. 2A, B). mRNA transcripts of OR1A1 were not detected (Fig. 2C). We concluded that OR1A1 is not expressed in AM and that Citronellal stimulates OR1A2. Therefore, OR1A1 was not further considered. The protein expressions of OR2AT4 and OR1A2 were confirmed by Western blot analyses. For each receptor we used two different primary antibodies in individual experiments for the validation of the patterns that are in line with published results from other cell types (Fig. 2D, E, Additional file 1: Fig. S2) (Jovancevic et al. 2017; Kalbe et al. 2017, 2016; Yang et al. 2015; Cook et al. 2009). The intracellular localization was analyzed using immunocytochemical staining. Overlay of the stainings for each OR with the staining for the plasma membrane marker E-cadherin suggested that both ORs are primarily expressed in the outer plasma membrane of AM (Fig. 2F, G).

### Sandalore increased the intracellular calcium concentration in AM via a cAMP-dependent pathway

Next, we investigated whether OR stimulation might influence pathophysiologically relevant intracellular processes. The calcium balance is disturbed in COPD and cystic fibrosis (Rimessi et al. 2021; Petit et al. 2019). Both, Sandalore and Brahmanol, are OR2AT4 agonists but the responses to Sandalore were more robust than those to Brahmanol (data not shown). Therefore, we used Sandalore to investigate the downstream signaling cascade of OR2AT4 in AM. In concentration–response experiments, Sandalore induced Ca^2+^ influx at an EC_50_ of 190 µM (Additional file 1: Fig. S1). Therefore, 300 µM was used in the following experiments.

**Fig. 3:**
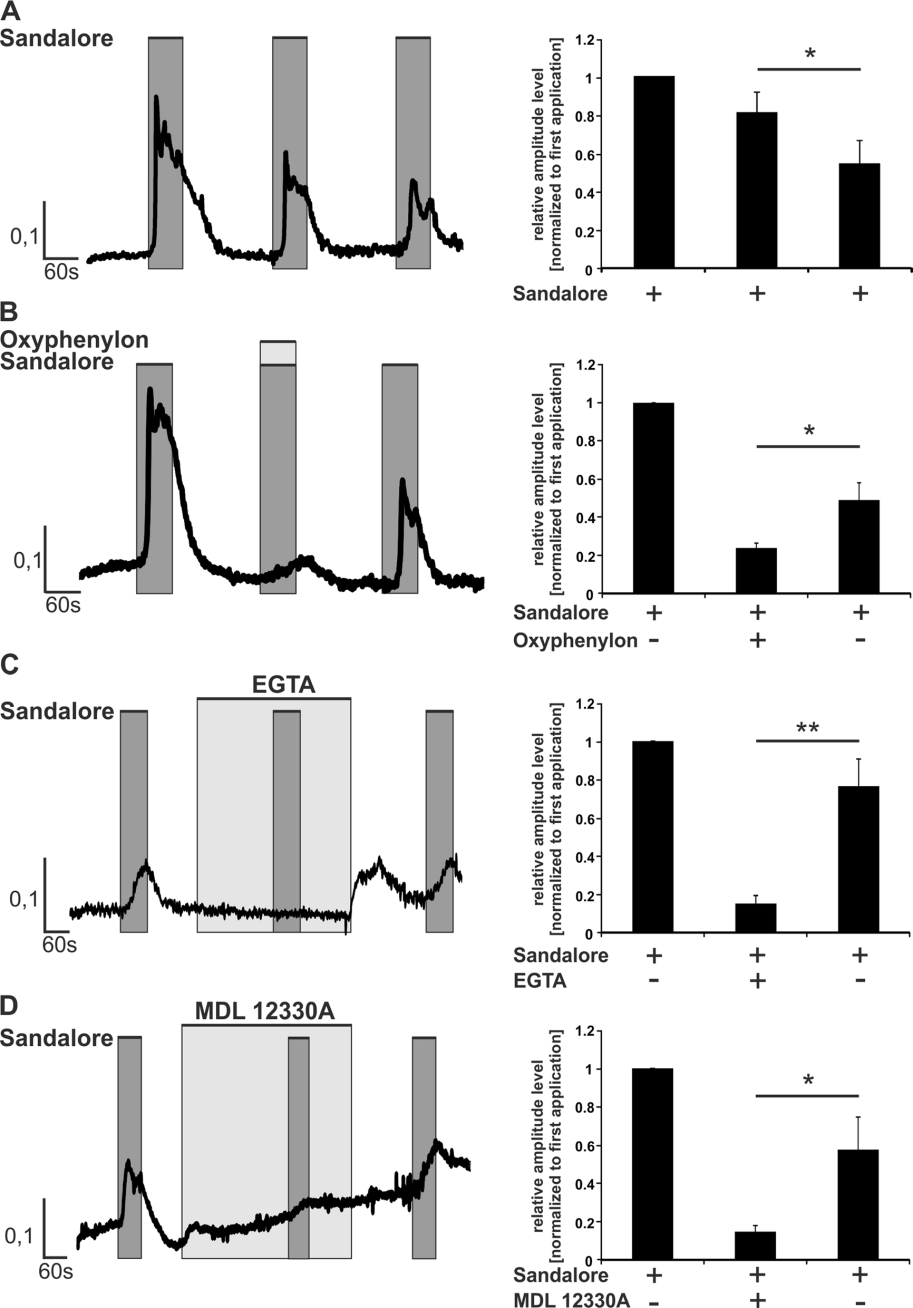
Sandalore mediates an increase of intracellular calcium via a cAMP-dependent pathway. AM were stimulated three times for 1 min each with 300 µM Sandalore (**A**, dark grey). Intermediary washing steps removed the odorant or inhibitor. **B** Repetitive application of 300 µM Sandalore with co-application of 300 µM Oxyphenylon (second application, light grey). EGTA 10 mM (**C**) and MDL-12,330A 10 μM (**D**) were pre-incubated for three (EGTA) or five minutes (MDL 12,330A) before adding Sandalore in the second application (light grey). Data are shown as mean ± SEM (n = 9 of 3 donors). Data were analyzed with the Student’s paired *t*-test referring to the third Ca^2+^ amplitude induced by Sandalore. *p ≤ 0.05 and **p ≤ 0.01

To investigate the signaling cascade that underlies the Ca^2+^-dependent response of AM to Sandalore, we used the following strategy based on highly selective pathway inhibitors. Sandalore was applied three times in a row with intermediary washing steps. The first and the third application were both in the absence (controls), and the second application was in the presence of the respective inhibitor. The second and third amplitudes of one experiment were normalized to the corresponding first amplitude to analyze for inhibitor’s effects. The repetitive application of Sandalore caused a desensitization of the increase of intracellular calcium (Fig. 3A), which was considered for analyzing the effects of the inhibitors by comparing the second with the third amplitude in each of the following experiments.

The OR2AT4 antagonist Oxyphenylon and the specific adenylate cyclase inhibitor MDL-12,300A (Busse et al. 2014; Guellaen et al. 1977; Simon et al. 1978), both reduced the Sandalore-induced intracellular calcium (Fig. 3B, D). To determine whether stimulation of OR2AT4 by Sandalore resulted in a calcium influx from extracellular or from a release from intracellular stores, the extracellular calcium was bound by 10 mM EGTA, which resulted in a substantial reduction of the amplitude (Fig. 3C). The third application of Sandalore in the absence of the respective inhibitors induced in each case more intracellular calcium than the second application in the presence of the inhibitors (Fig. 3B–D), demonstrating that the inhibitors did not affect cell viability.

**Fig. 4:**
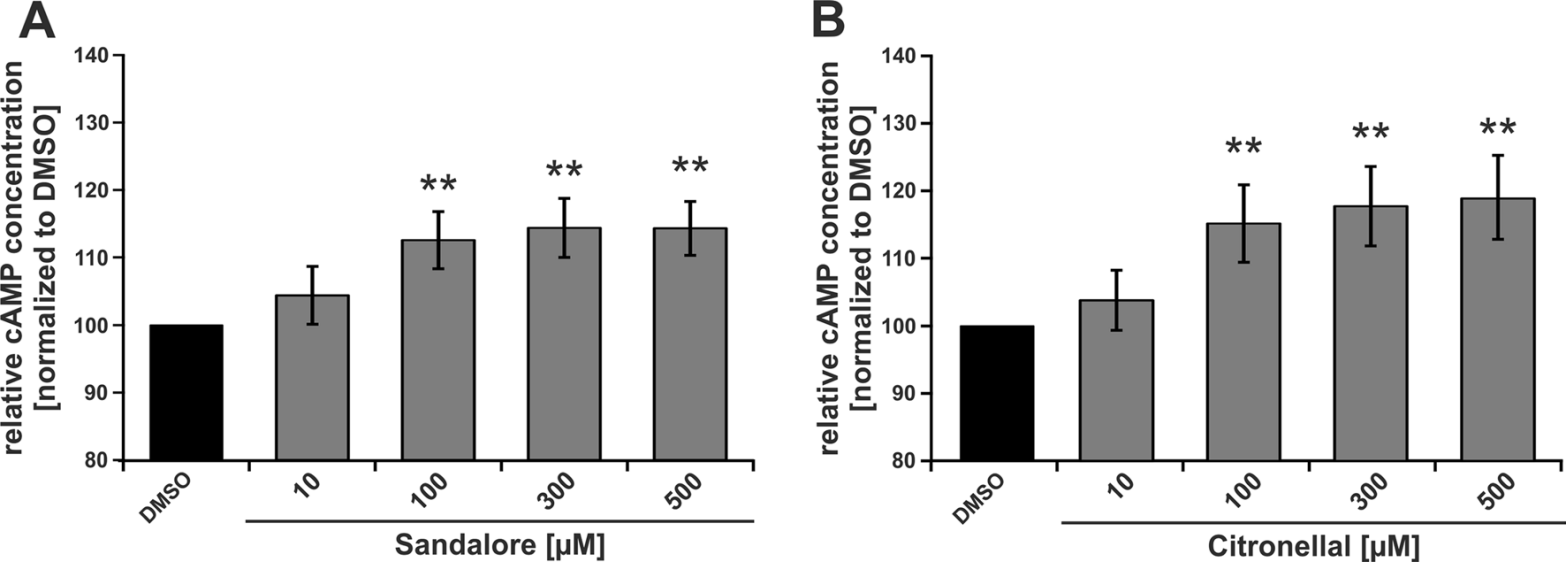
Sandalore and Citronellal increase the cAMP levels. AM were stimulated for 20 min with Sandalore (**A**) or Citronellal (**B**) at the indicated concentrations. The intracellular cAMP concentration was normalized to the solvent control (DMSO). Data (n = 5 donors) are presented as mean ± SEM and were analyzed using the Friedman test with post hoc “Two-stage-up method of Benjamini, Krieger, and Yekutieli,” referring to the solvent control. **p ≤ 0.01

Because of sample limitations, the experiments regarding downstream signaling for OR1A2 were only performed for the following endpoint experiments. Both, stimulation of OR2AT4 with Sandalore and stimulation of OR1A2 with Citronellal increased the intracellular cAMP levels in a concentration-dependent manner (Fig. 4).

### Sandalore and Citronellal both decreased the phagocytic activity of AM

**Fig. 5:**
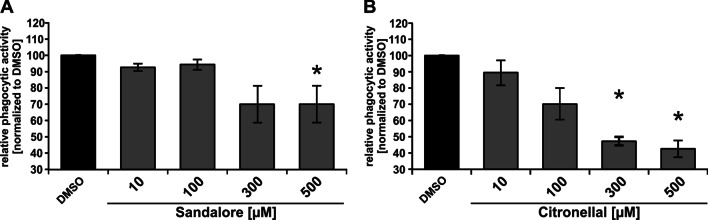
Sandalore and Citronellal reduce the phagocytic activity of AM. AM were incubated for two hours with fluorescent latex beads and Sandalore (**A**) or Citronellal (**B**). The amount of phagocytosed beads was determined by flow cytometry. Data were normalized to solvent controls (DMSO). Data (n = 3 donors) are presented as mean ± SEM and were analyzed using the Friedman test with post hoc “Two-stage-up method of Benjamini, Krieger, and Yekutieli,” referring to the solvent control. *p < 0.05

Phagocytosis of AM is impaired in COPD, cystic fibrosis, and non-type 2 asthma (Belchamber et al. 2019; Vandivier et al. 2009; Veen et al. 2020). Both, Sandalore and Citronellal significantly reduced the phagocytic activity of AM in a concentration-dependent manner (Fig. 5).

### Sandalore and Citronellal, both decreased the secretion of proinflammatory cytokines

The secretion of proinflammatory cytokines from AM contributes to the progression of COPD, cystic fibrosis, and severe non-type 2 asthma. The effects of the odorants were investigated in the absence and presence of bacterial PAMPs (pathogen-associated molecular patterns) that mimic bacterial infections in this cell culture model.

**Fig. 6:**
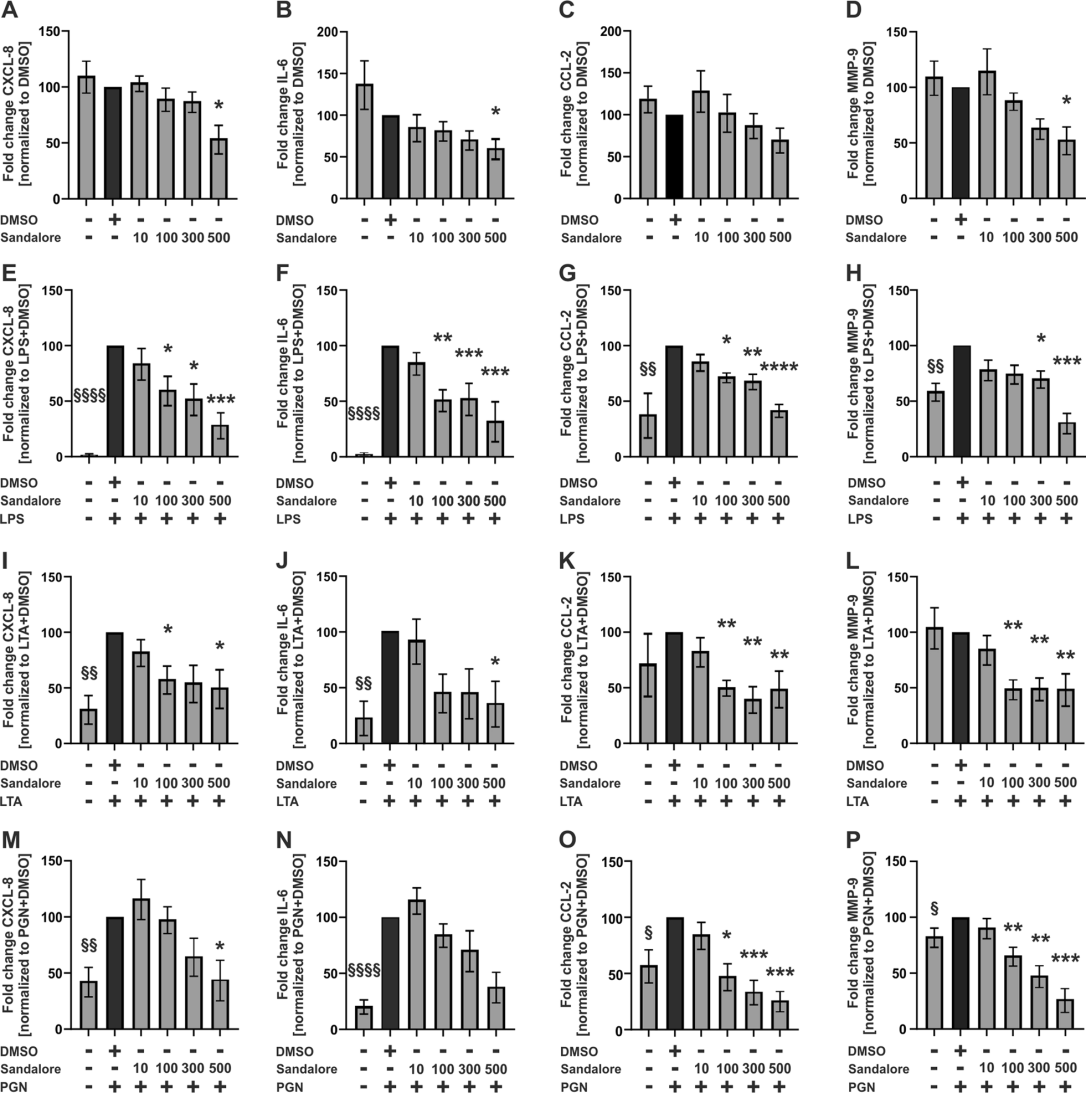
Sandalore reduces the secretion of CXCL-8, IL-6, CCL-2, and MMP-9 from alveolar macrophages. AM were stimulated with Sandalore (10–500 µM) and LPS (1 µg/ml), LTA (10 µg/ml), or PGN (10 µg/ml) for 24 h. CXCL-8, IL-6, CCL-2, and MMP-9 were measured in culture supernatants by ELISA. Data were normalized to solvent control (DMSO, **A**–**D**) or solvent control with LPS (**E**–**H**), LTA (**I**–**L**), or PGN (**M**–**P**). Data are shown as mean ± SEM n = 6–7 donors. A one-sample t-test analyzed cytokine induction by PAMPs compared to the solvent controls. ^§^p ≤ 0.05; ^§§^p ≤ 0.01, and ^§§§§^p ≤ 0.0001. Effects of Sandalore were analyzed by using the Friedman test with post hoc “Two-stage-up method of Benjamini, Krieger, and Yekutieli.” *p ≤ 0.05, **p ≤ 0.01, ***p ≤ 0.001, and ****p ≤ 0.0001

**Fig. 7:**
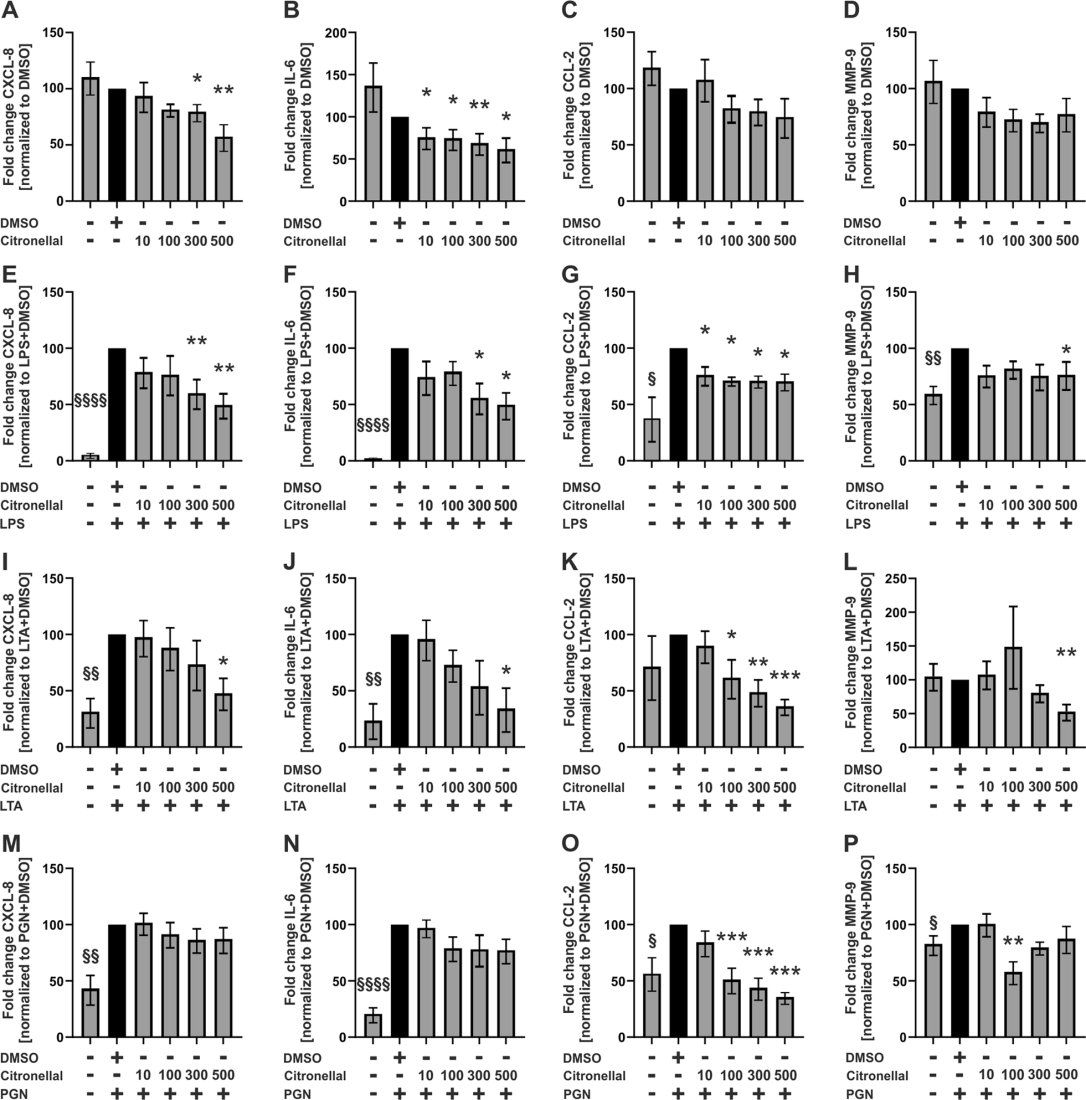
Citronellal reduces the secretion of CXCL-8, IL-6, CCL-2, and MMP-9 from alveolar macrophages. AM were stimulated with Citronellal (10–500 µM) and LPS (1 µg/ml), LTA (10 µg/ml), or PGN (10 µg/ml) for 24 h. CXCL-8, IL-6, CCL-2, and MMP-9 were measured in culture supernatants by ELISA. Data were normalized to solvent control (DMSO, **A**–**D**) or solvent control with LPS (**E**–**H**), LTA (**I**–**L**), or PGN (**M**–**P**). Data are shown as mean ± SEM n = 6–7 donors. A one-sample t-test analyzed cytokine induction by PAMPs compared to the solvent controls. ^§^p ≤ 0.05; ^§§^p ≤ 0.01, and ^§§§§^p ≤ 0.0001. Effects of Citronellal were analyzed by using the Friedman test with post hoc “Two-stage-up method of Benjamini, Krieger, and Yekutieli.” *p ≤ 0.05, **p ≤ 0.01, and ***p ≤ 0.001

Both Sandalore and Citronellal reduced baseline CXCL-8 and IL-6, but not CCL-2, Sandalore but not Citronellal reduced MMP-9 (Figs. 6A–D, 7A–D). LPS induced CXCL-8, IL-6, CCL-2, and MMP-9, which were reduced by Sandalore (Fig. 6E–H) and Citronellal (Fig. 7E–H).

LTA induced CXCL-8 and IL-6 (Figs. 6I, J, 7I, J) but not CCL-2 and MMP-9 (Figs. 6K, L, 7K, L). Both, Sandalore and Citronellal reduced LTA-induced CXCL-8 and IL-6 (Figs. 6I, J, 7I, J). Both odorants also reduced CCL-2 and MMP-9 in the presence of LTA (Figs. 6K, L, 7K, L).

PGN induced CXCL-8, IL-6, CCL-2, and MMP-9 (Figs. 6 M–P, 7M–P). Sandalore statistically significantly reduced PGN-induced CCL-2 and MMP-9 but not CXCL-8 and IL-6, although the data showed a strong trend in both cases (Fig. 6M–P). Citronellal reduced PGN-induced CCL-2 and MMP-9 but not CXCL-8 and IL-6 (Fig. 7M–P).

## Discussion

This study shows the functional expression of ORs in primary human AM for the first time. AM play a key role in the development and progression of chronic respiratory diseases with local non-type 2 inflammation like COPD, cystic fibrosis, and non-type 2 asthma (1) by releasing massive amounts of proinflammatory cytokines, (2) through misregulated phagocytotic activity, and (3) by recruiting and activating other immune cells (Balhara and Gounni 2012; Courtney et al. 2004; Barnes 2004). This study aimed to investigate whether ORs in AM might interfere with pathophysiological processes and thus might indicate as suitable drug targets for future treatment strategies for chronic respiratory diseases with AM-dependent inflammation. In contrast to respiratory diseases based on type 2 inflammation, current causal therapeutic options for macrophage-dependent inflammation are rare. One common strategy to discover functionally expressed ORs is to screen the target cells with their specific ligands via calcium imaging because most ORs regulate the intracellular calcium concentration (Jovancevic et al. 2017; Wojcik et al. 2018). In AM, stimulation with Sandalore, Brahmanol, and Citronellal induced a transient increase of the intracellular calcium level. Previous studies have already demonstrated that Sandalore and Brahmanol are agonists of OR2AT4, for example, by using the competitive antagonist Oxyphenylon (Busse et al. 2014). We have verified the data in the present study for AM. Citronellal stimulates OR1A1 and OR1A2 (Maßberg et al. 2015). Because OR1A2 transcripts and protein but no OR1A1 mRNA transcripts were detected in AM, we conclude that Citronellal stimulates OR1A2 in AM.

Besides OR1A2, OR2AT4 transcripts and proteins were also detected in AM. Most ORs, including OR1A2 and OR2AT4, have an estimated molecular weight of around 35 kDa (Kalbe et al. 2016; Belloir et al. 2017). Our Western blots with custom-made antibodies showed in each case signals at approximately 40 kDa. Increased molecular weights compared to theoretical estimations are not uncommon for ORs because of posttranslational modifications, as shown previously multiple times (Chéret et al. 2018; Zhao and Firestein 1999). Additional signals were also seen at sizes of 55, 70, and 100 kDa. Multiple previous studies indicated that these signals might represent multimers of the ORs (Chéret et al. 2018; Jovancevic et al. 2017; Yang et al. 2015). Particularly the additional signals at 55 or 70 kDa are prominent for several ORs (Jovancevic et al. 2017; Kalbe et al. 2017; Yang et al. 2015). We verified the Western blot patterns for both receptors by using alternative antibodies that are commercially available. Therefore, we can exclude that the signals are unspecific.

The expression of OR2AT4 and OR2J3 in AM is in line with findings showing the extranasal expression of several ORs in various cell types (Flegel et al. 2013; Lee et al. 2019). AM are located in the alveolar epithelial recess in the peripheral lung (Hussell and Bell 2014). Our data indicate that the two ORs are predominantly expressed in the outer plasma membranes, as described for the nasal epithelium and other tissues before (Kalbe et al. 2016; Bush and Hall 2008). Thus, putative inhaled drugs might have easy access to the ORs in AM, suggesting them as interesting target candidates regarding the route of drug delivery (Hardy and Chadwick 2000).

The downstream pathways need to be considered regarding a potential utility as drug targets. OR2AT4 and OR1A2 likely regulate the same pathways. Due to the limited amounts of samples, the signaling pathway analysis was performed in detail for OR2AT4 and was verified on key points for OR1A2. Our data provide evidence that both OR2AT4 and OR1A2 trigger a cAMP-dependent pathway leading to the activation of adenylate cyclase resulting in an influx of extracellular calcium. cAMP-dependent Ca^2+^-influx has previously been suggested as a promising therapeutic target to control steroid-resistant inflammation in COPD, non-type 2 asthma, and other chronic respiratory diseases with predominantly non-type 2 inflammation (Tintinger et al. 2005). For COPD, it has been demonstrated before that the production of the critical proinflammatory factor CXCL-8 from AM is insensitive to steroids (Barnes 2004; Khalaf et al. 2017). This might be key to the resistance to inhaled steroids in most COPD phenotypes. Mechanistically, this has been explained by a decreased expression and activity of histone-deacetylase-2 (HDAC2) in AM of COPD subjects that lead to a failure of activated glucocorticoid receptors to block the activity of the transcription factor NF-κB. NF-κB controls the transcription of CXCL-8 and other proinflammatory cytokines with central roles in non-type 2 inflammation and associated chronic respiratory diseases, including COPD (Barnes 2004, 2009). Here, we have demonstrated that the stimulation of two ORs reduces the expression of CXCL8 and other NF-κB-dependent and disease-related proinflammatory cytokines in AM likely via cAMP signaling. cAMP is known to counteract the release of NF-κB-dependent proinflammatory cytokines (Ivanov et al. 1997) by stimulating protein kinase A (PKA) (Minguet et al. 2005). Here, the transcription factor cAMP response element-binding protein (CREB) is phosphorylated via PKA and competes with NF-κB for the binding site of the coactivator CREB-binding protein (CBE) (Parry and Mackman 1997). In addition, cAMP prevents the phosphorylation of pERK and dephosphorylates p38 mitogen-activated protein kinase (Keränen et al. 2017), both of which are decisively involved in the secretion of proinflammatory cytokines in AM (Carter et al. 1999). Therefore, our data provide the first indication that the activation of cAMP signaling by OR stimulation might be an auspicious strategy to reduce or neutralize steroid-resistant non-type 2 inflammatory processes in the lung therapeutically. This might be considered for COPD and other chronic respiratory diseases with local AM-dependent and steroid-resistant non-type 2 inflammation like non-type 2 severe asthma phenotypes or cystic fibrosis.

The phagocytotic activity of macrophages is reduced in the lungs of COPD and cystic fibrosis subjects even though the AM numbers are increased (Ballinger et al. 2010; Jubrail et al. 2017; Nunes and Demaurex 2010; Lévêque et al. 2017). The reduced phagocytic activity might cause an ineffective clearing of bacterial infections of the respiratory tract, which can contribute to exacerbations or chronic infections (Lévêque et al. 2017; Donnelly and Barnes 2012). In severe asthma, the data are controversial, independent of the phenotype and the type of inflammation. Increased as well as decreased phagocytotic activity has been reported (Veen et al. 2020). Sandalore and Citronellal both suppressed the phagocytotic activity in AM. This could be explained by the transient increase of intracellular cAMP concentrations, which are known to inhibit phagocytosis (Ballinger et al. 2010; Nunes and Demaurex 2010). This effect might be based on the activation of the exchange proteins directly activated by cAMP (Epac), which has been shown to mediate the reduction of phagocytotic activity upon activation of other G-proteins (Aronoff et al. 2005; Steininger et al. 2011; Kittl et al. 2019). In contrast, Provost et al. showed that calcium restores the reduced phagocytic activity in patients with COPD (Provost et al. 2015). Effective phagocytosis of *Staphylococcus aureus* and other respiratory tract bacteria requires the activation of the transcription factor NF-κB (Zhu et al. 2014). NF-κB is inhibited by Ca^2+^/cAMP-dependent PKA (Minguet et al. 2005; Takahashi et al. 2002), which could be another mechanism through which Sandalore and Citronellal reduce phagocytotic activity in AM.

Regarding the potential of OR2AT4 and OR1A2 as putative drug targets, we conclude that stimulation of these receptors with the aim to neutralize local non-type 2 inflammation could be at the expense of further reducing the phagocytotic activity AM. Nonetheless, further studies might focus on which phenotypes or disease stages subjects might benefit more from reducing the inflammatory burden than maintaining the current phagocytotic activity of local cells. Infection-induced exacerbations and inflammation-induced remodeling of the lung tissue promote disease progression. It is conceivable that phenotypes with a low exacerbation frequency might profit from a strategy based on ORs as drug targets because their inflammation might be more important than misregulated phagocytosis. Whether these thoughts might also apply to a putative use in severe non-type 2 asthma remains unclear because of the controversial data situation about the misregulation of phagocytosis in these disease phenotypes. The experiments were performed with samples from patients with heterologous diseases. Therefore, the potential for individual disease phenotypes could not be fully elucidated.

Mechanistically, exacerbations might not only result from a defective clearance of bacteria but also from a substantial increase in inflammation. The latter might depend on an excessive response of AM to bacterial PAMPs regarding the release of proinflammatory cytokines and proteases like CXCL-8, CCL-2, and MMP-9 (Knobloch et al. 2011b). Therefore, the effect of OR stimulation on these key factors for non-type 2 respiratory diseases in the presence of bacterial PAMPs was investigated. We also considered IL-6, a marker whose production is rather reduced in COPD and cystic fibrosis in response to PAMPs (Knobloch et al. 2011b; Armstrong et al. 2009; John et al. 2010).

Stimulation of AM with LPS, the most important PAMP from gram-negative bacteria, resulted in a more robust cytokine secretion than with PGN and LTA, which are PAMPs from gram-positive bacteria. Sandalore and Citronellal both inhibited the secretion of CXCL-8, IL 6, CCL-2, and/or MMP-9 in the presence of the three PAMPs. This might be useful to reduce inflammation in exacerbations because CXCL-8, CCL-2, and MMP-9 are key factors and/or are hyper-produced in AM in response to bacteria (Knobloch et al. 2011b; Henrot et al. 2019). IL-6 might not be upregulated in the respiratory tract of COPD patients during acute infections. Indeed, there is evidence that IL-6 production in AM in response to bacteria is reduced, e.g. in COPD (Knobloch et al. 2011b; Armstrong et al. 2009). We nonetheless carefully conclude that ORs might have potential as drug targets in exacerbations induced by gram-negative or gram-positive bacterial infections. However, the consequence of IL-6 suppression should be addressed in further pre-clinical studies. There is an indication that—in contrast to local and resident AM—systemic defects in circulating immune cells might prevent their full cytokine response to respiratory bacteria at recruitment to the site of infection and activation in patients with smoking-induced non-type 2 inflammation (Knobloch et al. 2019). This might be a molecular explanation for the increased susceptibility to respiratory tract infections, e.g. in COPD (Rimessi et al. 2021; Petit et al. 2019). Therefore, it has to be investigated in future studies how ORs might influence cytokine expression in circulating immune cells in order to further evaluate their utility as drug targets in stages of infection-induced exacerbations.

Our study has some limitations. The cellular responses to ORs might be influenced by the inflammatory environment of the AM before isolation. The donors have not been phenotyped for the type of inflammation. However, according to the diagnosed diseases (please refer to Additional file 1: Table S1) and to the smoking status [smoking induces non-type 2 inflammation (Vaart et al. 2004; Arnson et al. 2010)] the presence of a partial or full respiratory non-type 2 inflammation was expected for all donors (Arnson et al. 2010) with on exception (see below). Active smoking and long-term ex-smoking asthmatics have an inflammatory profile with increased local and systemic neutrophils and a reduced steroid response compared to never-smoking asthmatics (Telenga et al. 2013). This indicates an involvement of non-type 2 inflammation in the pathogenesis of active and ex-smoking asthmatics (Hudey et al. 2020). Due to a lack of donors, we have included one never-smoker without a diagnosed respiratory disease and without indication for any kind of inflammation exclusively in the data for the Ca-dose response curve in Additional file 1: Fig. S1. However, it should be noted that the principle Ca-response of AM has also been shown in the data of Fig. 1, which included exclusively subjects with an indication for non-type 2 inflammation. Despite these limitations, our study clearly shows that ORs affect processes associated with non-type 2 inflammation in AM and it might be interesting to compare in further studies between patients with respiratory type 2, non-type 2, and mixed inflammatory phenotypes.

## Conclusion

In summary, our cell culture data provide the first indication for a possible utility of ORs in primary human AM as drug targets for respiratory diseases with AM-dependent non-type 2 chronic inflammation, such as COPD, cystic fibrosis, and non-type 2 asthma. In this context, the stimulation of OR2AT4 by Sandalore and OR1A2 by Citronellal impacts pathophysiologically misregulated processes like intracellular calcium levels, phagocytic activity, and inflammatory cytokine production in the presence and absence of bacterial PAMPs likely by a cAMP-dependent signaling pathway. Thus, our study promotes the idea of reducing steroid-resistant and AM-dependent inflammation via targeting Ca^2+^ signaling through ORs, a receptor class that might be easily reached by inhaled drugs. Because there is almost no therapy currently available for steroid-resistant non-type 2 inflammation in chronic lung diseases, future research should consider this possible strategy.

## Supplementary Information

**Additional file 1: Figure S1.** Sandalore increases the intracellular calcium concentration of human primary alveolar macrophages in a dose-dependent manner. **Figure S2.** Comparison of custom-made and commercially available antibodies against OR2AT4 and OR1A2. **Table S1.** Individual donor characteristics

## Data Availability

The original data and the analyses of the data that are presented in this study are available from the corresponding author on request.

## Abbreviations

AM: Alveolar macrophages
AP1: Activator protein 1
BAL: Bronchoalveolar lavage
Ca^2^^+^: Calcium
CCL: CC-chemokine ligand
cDNA: Complementary DNA
COPD: Chronic obstructive pulmonary disease
CXCL: CXC-motif-ligand
DMSO: Dimethylsulfoxide
DNA: Deoxyribonucleic acid
DTT: Dithiothreitol
EGTA: Ethylene glycol-bis(β-aminoethyl ether)-*N*,*N*,*N*′,*N*′-tetraacetic acid
GPCRs: G protein-coupled receptors
HDAC2: Histone-deacetylase-2
IL: Interleukin
LPS: Lipopolysaccharide
LTA: Lipoteichoic acid
MMP: Matrix metallopeptidase
NF-κB: Nuclear factor ‘kappa-light-chain-enhancer’ of activated B-cells
NSCLC: Non-small-cell lung cancer
ORs: Olfactory receptor
PAMPs: Pathogen-associated molecular patterns
PBS: Phosphate-buffered saline
PBST: Phosphate-buffered saline + Triton X-100
PCR: Polymerase chain reaction
PFA: Paraformaldehyde
PGN: Peptidoglycan
PKA: Protein kinase A
RPMI: Roswell Park Memorial Institute
RT-PCR: Reverse transcriptase PCR
SCLC: Small-cell lung cancer
SDS-PAGE: Sodium dodecylsulfate polyacrylamide gel electrophoresis
TBP: TATA-box binding protein

## References

[CR1] Abd El Qader A, Lieberman D, Shemer Avni Y, Svobodin N, Lazarovitch T, Sagi O (2015). Volatile organic compounds generated by cultures of bacteria and viruses associated with respiratory infections. Biomed Chromatogr.

[CR2] Adcock IM, Ford PA, Bhavsar P, Ahmad T, Chung KF (2008). Steroid resistance in asthma: mechanisms and treatment options. Curr Allergy Asthma Rep.

[CR3] Armstrong J, Sargent C, Singh D (2009). Glucocorticoid sensitivity of lipopolysaccharide-stimulated chronic obstructive pulmonary disease alveolar macrophages. Clin Exp Immunol.

[CR4] Arnson Y, Shoenfeld Y, Amital H (2010). Effects of tobacco smoke on immunity, inflammation and autoimmunity. J Autoimmun.

[CR5] Aronoff DM, Canetti C, Serezani CH, Luo M, Peters-Golden M (2005). Cutting edge: macrophage inhibition by cyclic AMP (cAMP): differential roles of protein kinase A and exchange protein directly activated by cAMP-1. J Immunol.

[CR6] Balhara J, Gounni AS (2012). The alveolar macrophages in asthma: a double-edged sword. Mucosal Immunol.

[CR7] Ballinger MN, Welliver T, Straight S, Peters-Golden M, Swanson JA (2010). Transient increase in cyclic AMP localized to macrophage phagosomes. PLoS ONE.

[CR8] Barnes PJ (2004). Alveolar macrophages as orchestrators of COPD. COPD.

[CR9] Barnes PJ (2009). Role of HDAC2 in the pathophysiology of COPD. Annu Rev Physiol.

[CR10] Beasley R, Semprini A, Mitchell EA (2015). Risk factors for asthma: is prevention possible?. Lancet.

[CR11] Belchamber KBR, Donnelly LE (2017). Macrophage dysfunction in respiratory disease. Results Probl Cell Differ.

[CR12] Belchamber KBR, Singh R, Batista CM, Whyte MK, Dockrell DH, Kilty I (2019). Defective bacterial phagocytosis is associated with dysfunctional mitochondria in COPD macrophages. Eur Respir J.

[CR13] Belloir C, Miller-Leseigneur M-L, Neiers F, Briand L, Le Bon A-M (2017). Biophysical and functional characterization of the human olfactory receptor OR1A1 expressed in a mammalian inducible cell line. Protein Expr Purif.

[CR14] Boucher RC (2019). Muco-obstructive lung diseases. N Engl J Med.

[CR15] Braun T, Voland P, Kunz L, Prinz C, Gratzl M (2007). Enterochromaffin cells of the human gut: sensors for spices and odorants. Gastroenterology.

[CR16] Bush CF, Hall RA (2008). Olfactory receptor trafficking to the plasma membrane. Cell Mol Life Sci.

[CR17] Busse D, Kudella P, Grüning N-M, Gisselmann G, Ständer S, Luger T (2014). A synthetic sandalwood odorant induces wound-healing processes in human keratinocytes via the olfactory receptor OR2AT4. J Invest Dermatol.

[CR18] Carter AB, Monick MM, Hunninghake GW (1999). Both Erk and p38 kinases are necessary for cytokine gene transcription. Am J Respir Cell Mol Biol.

[CR19] Castellani S, Di Gioia S, Di Toma L, Conese M (2018). Human cellular models for the investigation of lung inflammation and mucus production in cystic fibrosis. Anal Cell Pathol (Amst).

[CR20] Chen X, Li Y, Qin L, He R, Hu C (2021). Neutrophil extracellular trapping network promotes the pathogenesis of neutrophil-associated asthma through macrophages. Immunol Investig.

[CR21] Chéret J, Bertolini M, Ponce L, Lehmann J, Tsai T, Alam M (2018). Olfactory receptor OR2AT4 regulates human hair growth. Nat Commun.

[CR22] Chiu KY, Li JG, Lin Y (2017). Calcium channel blockers for lung function improvement in asthma: a systematic review and meta-analysis. Ann Allergy Asthma Immunol.

[CR23] Cook BL, Steuerwald D, Kaiser L, Graveland-Bikker J, Vanberghem M, Berke AP (2009). Large-scale production and study of a synthetic G protein-coupled receptor: human olfactory receptor 17-4. Proc Natl Acad Sci USA.

[CR24] Courtney JM, Ennis M, Elborn JS (2004). Cytokines and inflammatory mediators in cystic fibrosis. J Cyst Fibros.

[CR25] Culpitt SV, Rogers DF, Shah P, de Matos C, Russell REK, Donnelly LE (2003). Impaired inhibition by dexamethasone of cytokine release by alveolar macrophages from patients with chronic obstructive pulmonary disease. Am J Respir Crit Care Med.

[CR26] Cutting GR (2015). Cystic fibrosis genetics: from molecular understanding to clinical application. Nat Rev Genet.

[CR27] Donnelly LE, Barnes PJ (2012). Defective phagocytosis in airways disease. Chest.

[CR28] Duvall MG, Krishnamoorthy N, Levy BD (2019). Non-type 2 inflammation in severe asthma is propelled by neutrophil cytoplasts and maintained by defective resolution. Allergol Int.

[CR29] Enilari O, Sinha S (2019). The global impact of asthma in adult populations. Ann Glob Health.

[CR30] Fahy JV (2015). Type 2 inflammation in asthma—present in most, absent in many. Nat Rev Immunol.

[CR31] Flegel C, Manteniotis S, Osthold S, Hatt H, Gisselmann G (2013). Expression profile of ectopic olfactory receptors determined by deep sequencing. PLoS ONE.

[CR32] Garth J, Barnes JW, Krick S (2018). Targeting cytokines as evolving treatment strategies in chronic inflammatory airway diseases. Int J Mol Sci.

[CR33] Gelis L, Jovancevic N, Veitinger S, Mandal B, Arndt H-D, Neuhaus EM (2016). Functional characterization of the odorant receptor 51E2 in human melanocytes. J Biol Chem.

[CR34] Gerlach K, Köhler-Bachmann S, Jungck D, Körber S, Yanik S, Knoop H (2015). Endothelin receptor-antagonists suppress lipopolysaccharide-induced cytokine release from alveolar macrophages of non-smokers, smokers and COPD subjects. Eur J Pharmacol.

[CR35] Guellaen G, Mahu JL, Mavier P, Berthelot P, Hanoune J (1977). RMI 12330 A, an inhibitor of adenylate cyclase in rat liver. Biochim Biophys Acta.

[CR36] Hardy JG, Chadwick TS (2000). Sustained release drug delivery to the lungs: an option for the future. Clin Pharmacokinet.

[CR37] Hauser AS, Attwood MM, Rask-Andersen M, Schiöth HB, Gloriam DE (2017). Trends in GPCR drug discovery: new agents, targets and indications. Nat Rev Drug Discov.

[CR38] Henrot P, Prevel R, Berger P, Dupin I (2019). Chemokines in COPD: from implication to therapeutic use. Int J Mol Sci.

[CR39] Hikichi M, Mizumura K, Maruoka S, Gon Y (2019). Pathogenesis of chronic obstructive pulmonary disease (COPD) induced by cigarette smoke. J Thorac Dis.

[CR40] Hough KP, Curtiss ML, Blain TJ, Liu R-M, Trevor J, Deshane JS (2020). Airway remodeling in asthma. Front Med (Lausanne).

[CR41] Hudey SN, Ledford DK, Cardet JC (2020). Mechanisms of non-type 2 asthma. Curr Opin Immunol.

[CR42] Hussell T, Bell TJ (2014). Alveolar macrophages: plasticity in a tissue-specific context. Nat Rev Immunol.

[CR43] Ivanov VN, Lee RK, Podack ER, Malek TR (1997). Regulation of Fas-dependent activation-induced T cell apoptosis by cAMP signaling: a potential role for transcription factor NF-kappa B. Oncogene.

[CR44] Jartti T, Bønnelykke K, Elenius V, Feleszko W (2020). Role of viruses in asthma. Semin Immunopathol.

[CR45] John G, Yildirim AO, Rubin BK, Gruenert DC, Henke MO (2010). TLR-4-mediated innate immunity is reduced in cystic fibrosis airway cells. Am J Respir Cell Mol Biol.

[CR46] Jovancevic N, Khalfaoui S, Weinrich M, Weidinger D, Simon A, Kalbe B (2017). Odorant receptor 51E2 agonist β-ionone regulates RPE cell migration and proliferation. Front Physiol.

[CR47] Jubrail J, Kurian N, Niedergang F (2017). Macrophage phagocytosis cracking the defect code in COPD. Biomed J.

[CR48] Kalbe B, Knobloch J, Schulz VM, Wecker C, Schlimm M, Scholz P (2016). Olfactory receptors modulate physiological processes in human airway smooth muscle cells. Front Physiol.

[CR49] Kalbe B, Schulz VM, Schlimm M, Philippou S, Jovancevic N, Jansen F (2017). Helional-induced activation of human olfactory receptor 2J3 promotes apoptosis and inhibits proliferation in a non-small-cell lung cancer cell line. Eur J Cell Biol.

[CR50] Kapellos TS, Bassler K, Aschenbrenner AC, Fujii W, Schultze JL (2018). Dysregulated functions of lung macrophage populations in COPD. J Immunol Res.

[CR51] Keränen T, Hömmö T, Moilanen E, Korhonen R (2017). β2-receptor agonists salbutamol and terbutaline attenuated cytokine production by suppressing ERK pathway through cAMP in macrophages. Cytokine.

[CR52] Khalaf RM, Lea SR, Metcalfe HJ, Singh D (2017). Mechanisms of corticosteroid insensitivity in COPD alveolar macrophages exposed to NTHi. Respir Res.

[CR53] Khanjani S, Terzidou V, Johnson MR, Bennett PR (2012). NFκB and AP-1 drive human myometrial IL8 expression. Mediat Inflamm.

[CR54] Kiedrowski MR, Bomberger JM (2018). Viral-bacterial co-infections in the cystic fibrosis respiratory tract. Front Immunol.

[CR55] Kittl M, Jakab M, Steininger TS, Ritter M, Kerschbaum HH (2019). A swelling-activated chloride current in microglial cells is suppressed by Epac and facilitated by PKA—impact on phagocytosis. Cell Physiol Biochem.

[CR56] Knobloch J, Schild K, Jungck D, Urban K, Müller K, Schweda EKH (2011). The T-helper cell type 1 immune response to Gram-negative bacterial infections is impaired in COPD. Am J Respir Crit Care Med.

[CR57] Knobloch J, Hag H, Jungck D, Urban K, Koch A (2011). Resveratrol impairs the release of steroid-resistant cytokines from bacterial endotoxin-exposed alveolar macrophages in chronic obstructive pulmonary disease. Basic Clin Pharmacol Toxicol.

[CR58] Knobloch J, Panek S, Yanik SD, Jamal Jameel K, Bendella Z, Jungck D (2019). The monocyte-dependent immune response to bacteria is suppressed in smoking-induced COPD. J Mol Med (Berl).

[CR59] Koch A, Giembycz M, Stirling RG, Lim S, Adcock I, Wassermann K (2004). Effect of smoking on MAP kinase-induced modulation of IL-8 in human alveolar macrophages. Eur Respir J.

[CR60] Kõressaar T, Lepamets M, Kaplinski L, Raime K, Andreson R, Remm M (2018). Primer3_masker: integrating masking of template sequence with primer design software. Bioinformatics.

[CR61] Lambrecht BN, Hammad H (2013). Asthma: the importance of dysregulated barrier immunity. Eur J Immunol.

[CR62] Lara-Reyna S, Holbrook J, Jarosz-Griffiths HH, Peckham D, McDermott MF (2020). Dysregulated signalling pathways in innate immune cells with cystic fibrosis mutations. Cell Mol Life Sci.

[CR63] Lee S-J, Depoortere I, Hatt H (2019). Therapeutic potential of ectopic olfactory and taste receptors. Nat Rev Drug Discov.

[CR64] Lévêque M, Le Trionnaire S, Del Porto P, Martin-Chouly C (2017). The impact of impaired macrophage functions in cystic fibrosis disease progression. J Cyst Fibros.

[CR65] Licari A, Castagnoli R, Marseglia A, Olivero F, Votto M, Ciprandi G (2020). Dupilumab to treat type 2 inflammatory diseases in children and adolescents. Paediatr Drugs.

[CR66] Liu X, Yin S, Chen Y, Wu Y, Zheng W, Dong H (2018). LPS-induced proinflammatory cytokine expression in human airway epithelial cells and macrophages via NF-κB, STAT3 or AP-1 activation. Mol Med Rep.

[CR67] Manteniotis S, Wojcik S, Brauhoff P, Möllmann M, Petersen L, Göthert JR (2016). Functional characterization of the ectopically expressed olfactory receptor 2AT4 in human myelogenous leukemia. Cell Death Discov.

[CR68] Manteniotis S, Wojcik S, Göthert JR, Dürig J, Dührsen U, Gisselmann G (2016). Deorphanization and characterization of the ectopically expressed olfactory receptor OR51B5 in myelogenous leukemia cells. Cell Death Discov.

[CR69] Maßberg D, Hatt H (2018). Human olfactory receptors: novel cellular functions outside of the nose. Physiol Rev.

[CR70] Maßberg D, Simon A, Häussinger D, Keitel V, Gisselmann G, Conrad H (2015). Monoterpene (-)-citronellal affects hepatocarcinoma cell signaling via an olfactory receptor. Arch Biochem Biophys.

[CR71] Matucci A, Vivarelli E, Nencini F, Maggi E, Vultaggio A (2021). Strategies targeting type 2 inflammation: from monoclonal antibodies to JAK-inhibitors. Biomedicines.

[CR72] Mei D, Tan WSD, Wong WSF (2019). Pharmacological strategies to regain steroid sensitivity in severe asthma and COPD. Curr Opin Pharmacol.

[CR73] Minguet S, Huber M, Rosenkranz L, Schamel WWA, Reth M, Brummer T (2005). Adenosine and cAMP are potent inhibitors of the NF-kappa B pathway downstream of immunoreceptors. Eur J Immunol.

[CR74] Nunes P, Demaurex N (2010). The role of calcium signaling in phagocytosis. J Leukoc Biol.

[CR75] Osadnik CR, Singh S (2019). Pulmonary rehabilitation for obstructive lung disease. Respirology.

[CR76] Parry GC, Mackman N (1997). Role of cyclic AMP response element-binding protein in cyclic AMP inhibition of NF-kappaB-mediated transcription. J Immunol.

[CR77] Petit A, Knabe L, Khelloufi K, Jory M, Gras D, Cabon Y (2019). Bronchial epithelial calcium metabolism impairment in smokers and chronic obstructive pulmonary disease. Decreased ORAI3 signaling. Am J Respir Cell Mol Biol.

[CR78] Provost KA, Smith M, Arold SP, Hava DL, Sethi S (2015). Calcium restores the macrophage response to nontypeable haemophilus influenzae in chronic obstructive pulmonary disease. Am J Respir Cell Mol Biol.

[CR79] Quaderi SA, Hurst JR (2018). The unmet global burden of COPD. Glob Health Epidemiol Genom.

[CR80] Rimessi A, Pozzato C, Carparelli L, Rossi A, Ranucci S, de Fino I (2020). Pharmacological modulation of mitochondrial calcium uniporter controls lung inflammation in cystic fibrosis. Sci Adv.

[CR81] Rimessi A, Vitto VAM, Patergnani S, Pinton P (2021). Update on calcium signaling in cystic fibrosis lung disease. Front Pharmacol.

[CR82] Schmiedeberg K, Shirokova E, Weber H-P, Schilling B, Meyerhof W, Krautwurst D (2007). Structural determinants of odorant recognition by the human olfactory receptors OR1A1 and OR1A2. J Struct Biol.

[CR83] Schwartz MD, Moore EE, Moore FA, Shenkar R, Moine P, Haenel JB (1996). Nuclear factor-kappa B is activated in alveolar macrophages from patients with acute respiratory distress syndrome. Crit Care Med.

[CR84] Simon B, Dittrich J, Kather H, Encke A, Kommerell B (1978). Inhibition of human colonic adenylate cyclase by RMI 12330 A. Digestion.

[CR85] Soriano JB, Kendrick PJ, Paulson KR (2020). Prevalence and attributable health burden of chronic respiratory diseases, 1990–2017: a systematic analysis for the Global Burden of Disease Study 2017. Lancet Respir Med.

[CR86] Sriram K, Insel PA (2018). G protein-coupled receptors as targets for approved drugs: how many targets and how many drugs?. Mol Pharmacol.

[CR87] Stecenko AA, King G, Torii K, Breyer RM, Dworski R, Blackwell TS (2001). Dysregulated cytokine production in human cystic fibrosis bronchial epithelial cells. Inflammation.

[CR88] Steininger TS, Stutz H, Kerschbaum HH (2011). Beta-adrenergic stimulation suppresses phagocytosis via Epac activation in murine microglial cells. Brain Res.

[CR89] Stolz D, Papakonstantinou E, Grize L, Schilter D, Strobel W, Louis R (2019). Time-course of upper respiratory tract viral infection and COPD exacerbation. Eur Respir J.

[CR90] Takahashi N, Tetsuka T, Uranishi H, Okamoto T (2002). Inhibition of the NF-kappaB transcriptional activity by protein kinase A. Eur J Biochem.

[CR91] Telenga ED, Kerstjens HAM, ten Hacken NHT, Postma DS, van den Berge M (2013). Inflammation and corticosteroid responsiveness in ex-, current- and never-smoking asthmatics. BMC Pulm Med.

[CR92] Tintinger G, Steel HC, Anderson R (2005). Taming the neutrophil: calcium clearance and influx mechanisms as novel targets for pharmacological control. Clin Exp Immunol.

[CR93] Untergasser A, Cutcutache I, Koressaar T, Ye J, Faircloth BC, Remm M (2012). Primer3—new capabilities and interfaces. Nucleic Acids Res.

[CR94] van der Vaart H, Postma DS, Timens W, ten Hacken NHT (2004). Acute effects of cigarette smoke on inflammation and oxidative stress: a review. Thorax.

[CR95] van der Veen TA, de Groot LES, Melgert BN (2020). The different faces of the macrophage in asthma. Curr Opin Pulm Med.

[CR96] Vandivier RW, Richens TR, Horstmann SA, deCathelineau AM, Ghosh M, Reynolds SD (2009). Dysfunctional cystic fibrosis transmembrane conductance regulator inhibits phagocytosis of apoptotic cells with proinflammatory consequences. Am J Physiol Lung Cell Mol Physiol.

[CR97] Wang Y, Xu J, Meng Y, Adcock IM, Yao X (2018). Role of inflammatory cells in airway remodeling in COPD. Int J Chron Obstruct Pulmon Dis.

[CR98] Wang C, Zhou J, Wang J, Li S, Fukunaga A, Yodoi J (2020). Progress in the mechanism and targeted drug therapy for COPD. Signal Transduct Target Ther.

[CR99] Weber L, Al-Refae K, Ebbert J, Jägers P, Altmüller J, Becker C (2017). Activation of odorant receptor in colorectal cancer cells leads to inhibition of cell proliferation and apoptosis. PLoS ONE.

[CR100] Weber L, Schulz WA, Philippou S, Eckardt J, Ubrig B, Hoffmann MJ (2018). Characterization of the olfactory receptor OR10H1 in human urinary bladder cancer. Front Physiol.

[CR101] Weidinger D, Jovancevic N, Zwanziger D, Theurer S, Hönes J, Führer D (2021). Functional characterization of olfactory receptors in the thyroid gland. Front Physiol.

[CR102] Wojcik S, Weidinger D, Ständer S, Luger T, Hatt H, Jovancevic N (2018). Functional characterization of the extranasal OR2A4/7 expressed in human melanocytes. Exp Dermatol.

[CR103] Yang H, Song HS, Ahn SR, Park TH (2015). Purification and functional reconstitution of human olfactory receptor expressed in *Escherichia coli*. Biotechnol Bioprocess Eng.

[CR104] Zhao H, Firestein S (1999). Vertebrate odorant receptors. Cell Mol Life Sci.

[CR105] Zhu F, Yue W, Wang Y (2014). The nuclear factor kappa B (NF-κB) activation is required for phagocytosis of *Staphylococcus aureus* by RAW 264.7 cells. Exp Cell Res.

