# Supplementary material for: OR2AT4 and OR1A2 counterregulate molecular pathophysiological processes of steroid-resistant inflammatory lung diseases in human alveolar macrophages
Source: Mol Med. 2022 Dec 12;28:150. doi: 10.1186/s10020-022-00572-8 (PMC9743598; doi:10.1186/s10020-022-00572-8)
Supplement: Supplementary file 1 — Additional file 1: Figure S1. Sandalore increases the intracellular calcium concentration of human primary alveolar macrophages in a dose-dependent manner. Figure S2. Comparison of custom-made and commercially available antibodies against OR2AT4 and OR1A2. Table S1. Individual donor characteristics [file 10020_2022_572_MOESM1_ESM.docx]

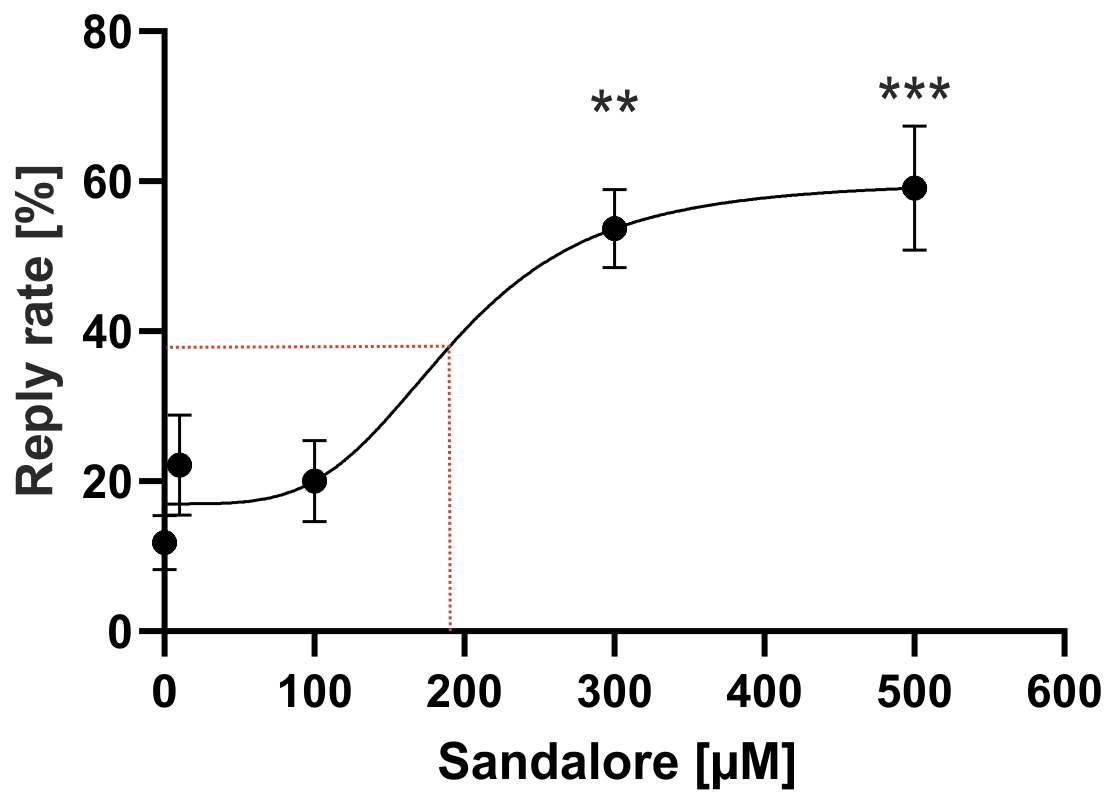


**Figure S1. Sandalore increases the intracellular calcium concentration of human primary alveolar macrophages in a dose-dependent manner.** The dose-response curve was fitted using Hill 3 calculation. The dotted line represents the EC_50_ value of 190 µM. Data significance was calculated by Friedman test with post hoc" Two-stage-up method of Benjamini, Krieger, and Yekutieli" referring to the solvent control. The mean values ± SEM are shown (n=9 of three donors), **p≤0.01, and ***p≤0.001.


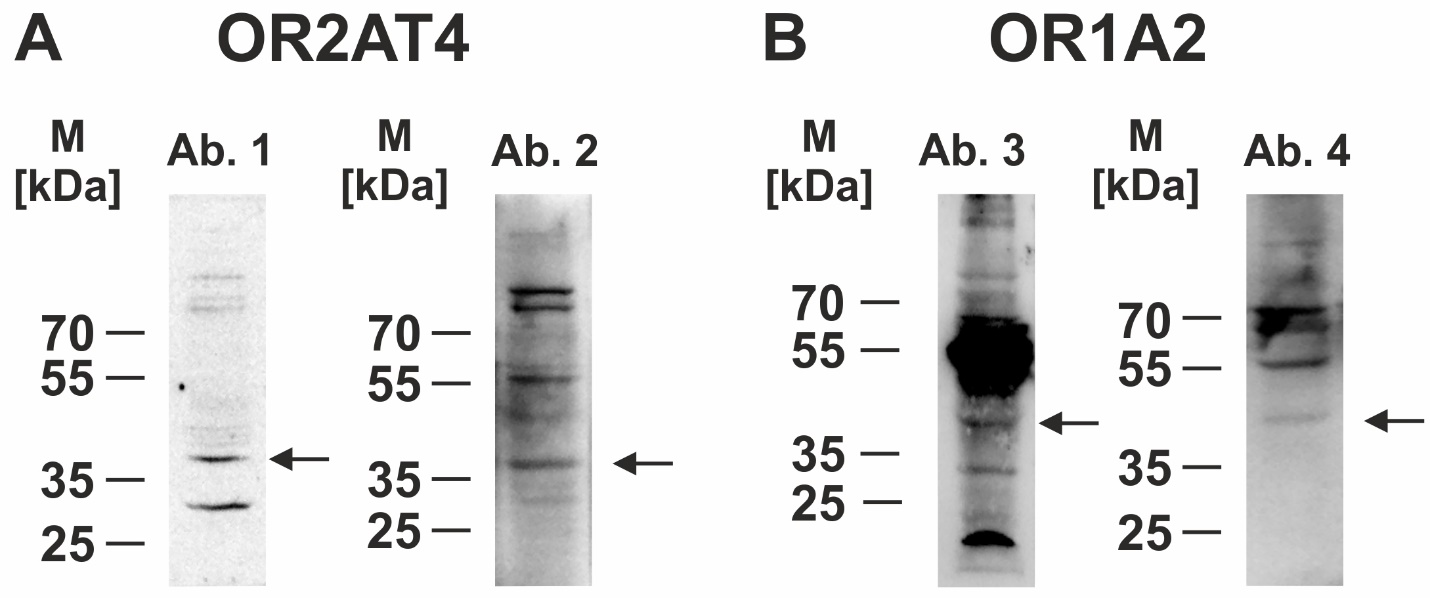


**Figure S2. Comparison of custom-made and commercially available antibodies against OR2AT4 and OR1A2.** Proteins of AM were prepared as given in the methods section. Custom-made affinity purified rabbit IgG polyclonal antibody (Ab.) against OR2AT4 or OR1A2 (Eurogentec; Ab 1 and 3) were compared with corresponding commercially available antibodies (Thermo Fisher Scientific, Ab. 2 and 4). Arrows indicate the OR monomers.

**Table S1: Individual donor characteristics.** Sex, age, diagnosed lung disease(s), smoking status, and the experimental use of the corresponding sample are listed. Non-pulmonary diseases were not considered. The severity of chronic obstructive pulmonary disease (COPD) was reported according to Standard level, I-IV. Calcium-Imaging (Ca-Img), Enzyme-linked Immunosorbent Assay (Elisa), Immunocytochemistry (ICC), non-small cell lung cancer (NSCLC), Polymerase chain reaction (PCR), Phagocytosis Assay (Phagocytosis), Western blot (WB).

| Patient number | Sex | Age | Diagnosed Lung Diseases | Smoking status | Experiment used for |
| --- | --- | --- | --- | --- | --- |
| 1 | Female | 63 | Severe asthma | Ex-Smoker | Ca-Img |
| 2 | Male | 61 | COPD Gold IV | Active smoker | Ca-Img |
| 3 | Female | 71 | Pulmonary space-occupying lesions | Ex-Smoker | PCR |
| 4 | Male | 80 | COPD Gold II | Active smoker | Ca-Img |
| 5 | Male | 70 | NSCLC, COPD Gold II | Active smoker | Ca-Img |
| 6 | Male | 82 | Silicosis | Active smoker | Ca-Img |
| 7 | Male | 44 | COPD Gold II | Active smoker | Ca-Img |
| 8 | Male | 42 | Sarcoidosis | Ex-Smoker | PCR |
| 9 | Male | 62 | Sarcoidosis | Ex-Smoker | Ca-Img |
| 10 | Male | 69 | NSCLC | Active smoker | Ca-Img |
| 11 | Female | 59 | Asthma-COPD-Overlap Syndrom | Active smoker | Ca-Img |
| 12 | Male | 68 | COPD Gold III | Active smoker | WB |
| 13 | Male | 89 | COPD Gold I, Silicosis | Active smoker | Dose response curve |
| 14 | Male | 77 | COPD Gold III | Active smoker | WB |
| 15 | Female | 80 | None | Never smoker | Dose response curve |
| 16 | Male | 72 | Chronic bronchitis | Ex-Smoker | ICC |
| 17 | Male | 54 | None | Active smoker | Dose response curve |
| 18 | Male | 84 | Lung nodule, COPD Gold III | Unknown | Ca-Img, ICC |
| 19 | Female | 57 | Interstitial Lung Disease | Active smoker | Ca-Img |
| 20 | Male | 79 | COPD Gold III | Active smoker | Ca-Img |
| 21 | Female | 85 | None | Active smoker | Ca-Img, ICC |
| 22 | Male | 60 | Alveolitis | Ex-Smoker | Phagocytosis |
| 23 | Male | 72 | COPD Gold III | Active smoker | Ca-Img |
| 24 | Male | 77 | COPD Gold III, Sarcoidosis | Ex-Smoker | Phagocytosis |
| 25 | Male | 78 | COPD Gold II, NSCLC | Active smoker | Phagocytosis |
| 26 | Female | 63 | Space-occupying lesions | Active smoker | Elisa |
| 27 | Male | 86 | Pneumonia | Active smoker | Elisa |
| 28 | Male | 67 | None | Ex-Smoker | Elisa |
| 29 | Male | 87 | COPD Gold II, Pneumonia | Ex-Smoker | Elisa |
| 30 | Female | 54 | Asthma | Active smoker | Elisa |
| 31 | Male | 62 | Alveolitis | Ex-Smoker | Elisa |
| 32 | Male | 64 | COPD Gold II, Pneumonia, Space-occupying lesions | Active smoker | Elisa |
| 33 | Female | 68 | NSCLC, COPD | Ex-Smoker | Elisa |
| 34 | Male | 46 | Asthma | Active smoker | WB, PCR, Elisa |
| 35 | Female | 56 | Space-occupying lesions | Ex-Smoker | Elisa |
| 36 | Male | 50 | Asthma, Sarcoidosis, Asbestosis | Active smoker | Elisa, cAMP |
| 37 | Female | 72 | NSCLC | Ex-Smoker | Elisa, cAMP |
| 38 | Female | 64 | Interstitial Lung Disease, Pneumonia | Active smoker | Elisa, cAMP |
| 39 | Male | 48 | Respiratory failure, Ex Covid, Neutrophilia | Never smoker | WB, Elisa |
| 40 | Male | 75 | Space-occupying lesions | Ex-Smoker | Elisa |
| 41 | Male | 52 | Space-occupying lesions | Active smoker | Elisa |
| 42 | Male | 61 | Interstitial Lung Disease | Active smoker | cAMP |
| 43 | Male | 39 | Lung nodule | Active smoker | Elisa |
| 44 | Female | 56 | Chronic cough | Ex-Smoker | Elisa, cAMP |
| 45 | Male | 62 | Interstitial Lung Disease | Active smoker | Elisa |
| 46 | Male | 55 | Pneumonia | Active smoker | Elisa |
| 47 | Male | 66 | COPD IV | Active smoker | ICC |
| 48 | Male | 78 | NSCLC | Ex-Smoker | ICC |
| 49 | Male | 66 | Pneumonia | Ex-Smoker | ICC |
| 50 | Male | 48 | COPD | Never smoker | Elisa |
